# DEDDIAG, a domestic electricity demand dataset of individual appliances in Germany

**DOI:** 10.1038/s41597-021-00963-2

**Published:** 2021-07-15

**Authors:** Marc Wenninger, Andreas Maier, Jochen Schmidt

**Affiliations:** 1 Rosenheim Technical University of Applied Sciences, Dep. of Computer Science, 83024 Rosenheim, Germany; 2 Pattern Recognition Lab, Friedrich-Alexander-University Erlangen-Nürnberg, 91058 Erlangen, Germany

**Keywords:** Scientific data, Computational science, Software, Energy and behaviour, Energy supply and demand

## Abstract

Real-world domestic electricity demand datasets are the key enabler for developing and evaluating machine learning algorithms that facilitate the analysis of demand attribution and usage behavior. Breaking down the electricity demand of domestic households is seen as the key technology for intelligent smart-grid management systems that seek an equilibrium of electricity supply and demand. For the purpose of comparable research, we publish DEDDIAG, a domestic electricity demand dataset of individual appliances in Germany. The dataset contains recordings of 15 homes over a period of up to 3.5 years, wherein total 50 appliances have been recorded at a frequency of 1 Hz. Recorded appliances are of significance for load-shifting purposes such as dishwashers, washing machines and refrigerators. One home also includes three-phase mains readings that can be used for disaggregation tasks. Additionally, DEDDIAG contains manual ground truth event annotations for 14 appliances, that provide precise start and stop timestamps. Such annotations have not been published for any long-term electricity dataset we are aware of.

## Abstract (summary)

**Table:** 

Measurement(s)	domestic appliance electricity usage • whole-house mains reading • ground truth event annotations
Technology Type(s)	energy measurement system • Annotation
Sample Characteristic - Environment	house
Sample Characteristic - Location	Germany

Machine-accessible metadata file describing the reported data: 10.6084/m9.figshare.14753556

## Background & Summary

For many years electricity consumption has only been monitored for billing purposes, thus only requiring metering devices that provide readings for each billing period. In recent years smart-meters have been installed to a greater extent to provide a basis for more complex pricing models as well as a more in-depth consumption analysis. The European Union has started this transformation as part of the *Third Energy Package* in the directive 2009/72/EC1^1^ in order to promote energy efficiency by “developing innovative pricing formulas, or introducing intelligent metering systems or smart grid”. The target was to have smart-meters installed in 80% of households by 2020 with the goal to improve customer awareness regarding electricity efficiency and increase stability and reliability of the grid^2^. While higher resolution information is the basis for more detailed usage analysis, the raw electricity consumption data will not provide any meaningful benefit as it lags a required abstraction as to what causes the electricity consumption, and, even more important, what could be done to alter the consumption. Enabling meaningful retrospective insights as well as prospective suggestions on consumer electricity consumption, mechanisms for information retrieval, behavior analysis, and forecasting have to be developed. The biggest enabler for research are datasets, and even more significant are publicly available datasets, since only then different research teams can evaluate publications and further develop established techniques. This is especially critical as the gathering of high resolution electricity consumption data is accompanied by many ethical questions, especially if not implemented as opt-in^3^.

Kolter and Johnson^4^ published the first dataset for electricity disaggregation. At the time they argued, that “although there are vast amounts of data relevant to energy domains the majority of this data is unavailable to researchers”. Furthermore, the authors argue that many other domains have greatly benefited from public benchmark data sets such as MNIST^5^ for handwritten digit recognition or PASCAL^6^ for visual object category recognition and detection.

Since then, more datasets have been released with different target electricity applications in mind^4,7–21^. A review on electricity datasets^22^ highlights the diversity of the datasets as they differ in location, duration, frequency, file format and many more aspects. The authors demand two primary objectives for the collection and provisioning of datasets, for (1) interoperability and (2) comparability. This would help to decrease heterogeneity, thus increase comparability of publications. An aspect highlighted by the authors is missing meta data describing the circumstances of the data recording. We found this to be especially relevant in order to be able to find explanations for behavior and behavior changes.

The DEDDIAG (domestic electricity demand dataset of individual appliances in Germany) dataset was mainly recorded in order to provide the basis for automated domestic load-shift applications where, e. g., a Real-Time-Price is used as an incentive. Hence, the dataset contains appliances that have the potential for automated load-shifting such as fridges, freezers, dishwashers, dryers and washing machines. According to the German Federal Environment Agency, other applications, outside of space heating and cooling, such as washing machines, dryers, stoves, refrigerators and similar, account for 52.6% of a household’s electricity demand in 2018^23^. It is uncommon to use electricity based space heating, and therefore space heating accounts only for 5.8%; space cooling systems are in general uncommon and only account for 1.0%^24^. In some cases additional appliances that may not contribute to load-shifting were recorded as they give insight into habits. We find it especially important to provide annotations for appliance usage in the form of Start-End annotations that attribute a certain time-span to a label. For instance, on a washing machine this is the cycle start and end combined with the program used; for a fridge this highlights the compressor cycles as well as the light that indicates an open fridge door. The latter is especially interesting as it gives a clear indication of an occupant being present.

We therefore not only publish the raw data, but also manual annotations, demographic information and model names for some appliances to provide a basis for research of device identification, behavior analysis and load-shift recommendation systems.

## Methods

In the following, an electricity data collection system for single appliances as well as whole-house level is described. The general requirements were derived from a 2 year load-shift monitoring project, where homes were monitored. The goal of the project was to record real-world behavior to understand load-shift potential, and at a later stage provide the homes with RTP (Real-Time-Pricing) incentives for load-shifting. As part of the project, a statistic based appliance usage prediction algorithm was developed to reduce the complexity of RTP behavior recommendations systems^25^. Further, an event segmentation algorithm for appliances identification in raw electricity measurements was developed^26^. The complete data collection software is available for download (see Sect. Code Availability for details).

### Requirements

The collection system was built with a focus on recording data for a user behavior change scenario such as Real-Time-Pricing (RTP). Therefore, the focus was on recording appliances with the following properties:

- easily shiftable load,
- significant electricity consumption,
- standard power plug.

Appliances falling under these terms are washing machines, dishwashers, fridges and freezers, thus the datasets mostly contain these appliances.

The general requirements for the project were:

- collect appliances and, optionally, whole-house electricity usage,
- store data locally,
- upload data frequently to central server,
- collect over a long time period (>2 years) at ≈1 Hz,
- no technical knowledge required to install the system,
- keep costs low.

#### Hardware requirements

The hardware requirements identified for the project were: The complexity of the hardware and software installation must be reduced so that a person without any special technical knowledge is able to install the system in their home. Therefore, the hardware for appliance-level measurements must be an end-user product that simply needs to be plugged in between wall-plug and appliance. The measuring plugs should support wireless communication to lower the complexity of the setup as the user otherwise would have to install cables in each room. For safety reasons, the installation of a house-level smart-meter must be performed by an electrician. Therefore, the technical knowledge required can be higher, but should also be kept as low as possible. The sample frequency for both appliance-level and home-level meter must be at least 1 Hz. This rate is used by a large number of algorithms already, following the suggestion of Klemenjak, C. *et al*.^22^ for macroscopic datasets. Higher sample rates increase the data handling complexity. It needs to be kept in mind that doubling the sample rate will also double the data volume that needs to be handled. Collected data must be stored on a small on-premises computer. The overall cost should be kept as low as possible.

#### Software development requirements

With a limited overall time for building and using the monitoring system, the goal for the development was to measure as early in the project as possible. This meant that system had to be installed in the homes as soon as possible. Therefore, it was developed in a MVP (Minimum Viable Product) fashion. The homes were distributed throughout the south of Germany and the project team did not have direct physical access to the homes.

The software requirements identified for the project were: The software system must be able to read the measurements from appliance- and home-meter and store it on an on-premises computer. The users should be provided with a web interface where they can monitor real-time meter values. All local data (client) must be uploaded hourly to a centralized server over the internet. The uploads must be self healing, meaning the upload system needs to be robust against a broken internet link, aborted/partial uploads and server downtime. The software must be able to be updated automatically in order to ship bug fixes and new features. For security reasons direct network access to the households network must be avoided. Thus the system must be updated autonomously without any interaction simply by providing a new software version on a server.

### System architecture

**Fig. 1:**
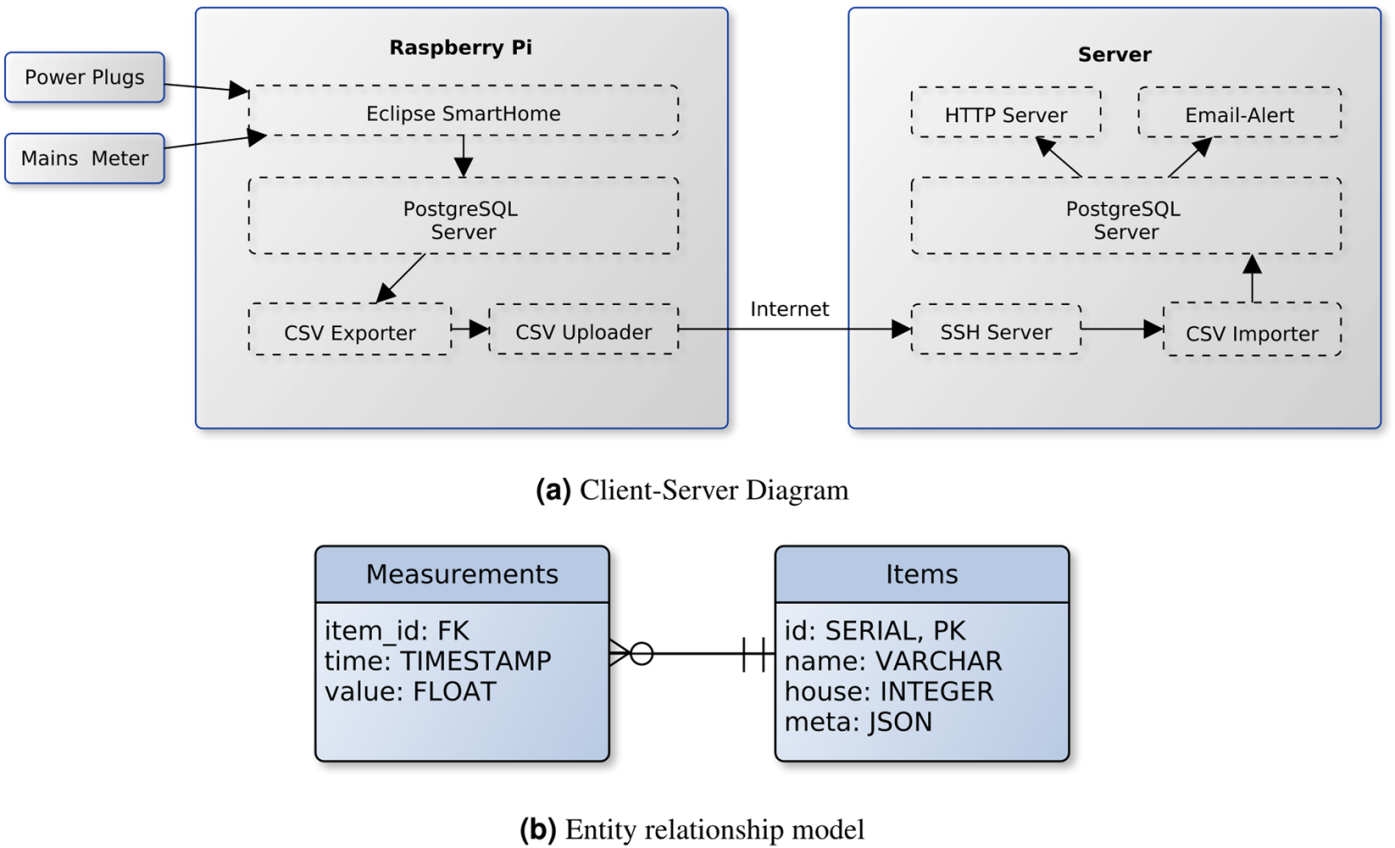
(**a**) Diagram showing detailed client-server components and data flow of the developed domestic energy measurement system. The Raspberry Pi (Client) is installed into the household collecting measurements meters. Data is persisted locally and automatically uploaded each our to a central server using the households internet connection. (**b**) Entity relationship model of server-side data storage.

Based on the requirements listed above, we derived the following system architecture (cf. Figure 1a). All components on server and client are designed as micro services and run as containers using Docker (https://www.docker.com). This enables a guaranteed software state on each client as well as a simple update mechanism.

#### Server-side

The server side provides three main functionalities: Data storage including secure upload, visualization, provide software updates, and monitoring. For data storage a PostgreSQL (https://www.postgresql.org) database is used, an open source relational database that provides a rich set of query functionality. The measurements of all homes and appliances are stored in a single table. In the following, all measurement sources, i. e. appliance or smart-meter phase, are called item. Each item is identified by a unique ID that is assigned on first upload of measurements to the server. All measurements are stored into a database scheme as shown in Fig. 1b, where all measurements are stored in a single table with a foreign-key to an items table. The items table contains the assigned unique ID as well as meta data such as item name and which household it is installed in.

As server-side hardware we used an Intel Xeon Gold 6140 CPU @ 2.30 GHz with 256 GB RAM. Such powerful hardware is in general not required, but increases SQL query speed when large number of measurements are recorded. It must be noted that in order to utilize the hardware power, the PostgreSQL instance needs to be configured to allocate more memory per query. The server requires an internet link capable of handling the uploaded data of about 140 KB per appliance per hour. This is insignificant compared to the required download capabilities. Daily backups and large queries require gigabytes to be transferred as fast as possible, and we therefore made use of a 1-Gbit internet link.

#### Home-/client-side

**Fig. 2:**
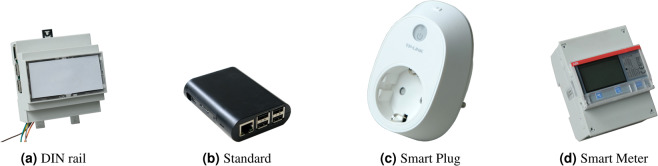
Choice of Raspberry Pi cases are shown on the left and choice of meters on the right. (**a**) is used for mounting on a DIN rail next to a whole-house meter, and (**b**) for all other setups. (**c**) is the smart plug used for individual appliance monitoring and (**d**) for three-phase mains monitoring.
- whole-house meter: ABB B23 112-100 + MAX485 module (see Fig. 2d),
- 5 V DC power supply.

The hardware components used for each home were:

- computer: Raspberry Pi 3 Model B with 32 GB storage,
- WiFi Access-Point: TP-Link TL-WR802N,
- individual appliance meter: TP-Link SmartPlug HS110 (see Fig. 2c),

For the on-premises computer a Raspberry Pi 3 Model B with 32 GB storage with a standard casing (see Fig. 2b) was used. It offers WiFi, Ethernet and GPIOs for Modbus communication with a whole-house meter at very low cost. In cases where a whole-house meter was installed, a DIN rail casing as shown in Fig. 2a was used to mount the Pi next to the meter as the read out using Modbus requires a cable connection.

The computer runs Hypriot OS (https://blog.hypriot.com), a slim operating system with the main focus on Docker container support. The system is based on Eclipse Smarthome (https://github.com/eclipse-archived/smarthome), which is used for collecting and persisting the data locally into a PostgreSQL database. The appliance meters are read over the wireless network every second, where the timestamp is added at receiving time of the value from the meter, thus introducing a reading latency equivalent to response time of device plus network latency. The system time is set using the NTP protocol. In order to keep the 1 Hz rate, the next value reading request takes this latency into account. Readings are persisted only on value change to minimize storage space. Additionally, one value is always stored at the full hour in order to detect connectivity problems.

Each hour the new data is exported into a CSV file and uploaded to the central server using SSH with key authentication. In order to identify incomplete uploads on server side, the SHA checksum is used as a filename and checked before import on the server side.

The full system can be pre-configured in a lab. This reduces the steps required for installation by the residents in each home to: Plug in individual appliance meters and Raspberry Pi, and connect the access point to a local router. This also allows to ship the system as a package without requiring an electrician when only appliance data is collected (i. e., no whole-house meter installed).

### Event annotations

The collection of raw data is an important step of all electricity machine learning efforts as it can act as ground truth. For some applications such as appliance usage analysis, the raw electricity measurements are only one part of the required ground truth, as electricity consumption does not necessarily correlate with usage. While the BLUED^9^ dataset does provide ground truth event annotation, the dataset cannot be used for tasks such as user behavior analysis as the recorded period is about 12 hours.

**Fig. 3:**
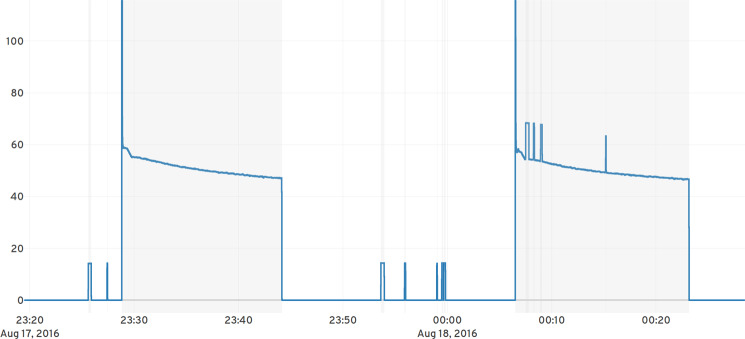
Measurements (blue curve) and annotations (light gray) of a refrigerator (House 5/Appliance 09) showing annotations for compressor and light cycles over a period of 1.5 hours on August 17th, 2016. Compressor and light are annotated as a separate event in order to classify user interaction (door opening) by using the light annotations.

Pereira^27^ takes the annotations from BLUED, adds manual annotations to the UK-DALE dataset and combines the results into a new event annotation dataset called NILMPEds. In this non-intrusive load monitoring context (NILM) events usually are defined as switch-on or off using a single timestamp. These two events have a logical relationship as a switch-on/start must be followed by a switch-off/stop. We therefore publish manual annotations for some of our data where an event is defined by two timestamps: ${e}_{0}=({t}_{0},\;{t}_{1})$ where ${t}_{0} < {t}_{1}$. Manual annotations were created using expert knowledge in a tool that allows multiple people to annotate data at the same time. Figure 3 shows manual annotations of compressor and light for a refrigerator in house 5. The measurements in the annotation tool have been rounded to full seconds using the provided SQL function round_timestamp() and the annotations are therefore created to full seconds.

## Data Records

We provide raw 1 Hz power readings of load-shifting relevant appliances over periods of 21 to 1351 days. For one home (house 8) we also provide whole-house mains readings. In total, the dataset contains 15 homes with a total of 50 appliances. A detailed listing of all homes and appliances, duration and missing data can be found in Table 1.

**Table 1:** List of houses and appliances observed in the DEDDIAG dataset.

Item	Category	Type	First date	Last date	Duration	Missing >1h5sec	Missing >1day
**House 0**							
10	Refrigerator		2016-11-30 20:24:05	2019-06-02 17:56:17	913 days	19.29%	7.85%
**House 1**							
1	Refrigerator		2016-10-06 19:25:07	2017-04-11 16:00:06	186 days	7.44%	2.91%
2	Washing Machine		2017-02-18 15:01:05	2017-04-11 15:00:00	51 days	22.17%	2.80%
4	Dish Washer	Bosch	2016-10-06 20:18:07	2017-04-11 16:00:04	186 days	59.40%	49.26%
**House 2**							
11	Freezer		2016-12-08 15:21:13	2020-08-20 22:00:07	1351 days	6.85%	6.76%
12	Washing Machine		2016-12-08 15:24:12	2018-12-12 14:43:51	733 days	14.73%	11.30%
**House 3**							
13	Freezer	Miele F 12020 S-3	2017-01-07 09:57:15	2018-05-27 07:59:33	504 days	2.50%	2.07%
14	Washing Machine	Bosch Maxx 6 ecoSpar	2016-12-20 09:18:54	2019-07-13 10:00:00	935 days	6.57%	3.34%
16	Refrigerator	Miele K14827 SD ED/CS	2017-01-07 09:57:15	2019-07-13 10:00:07	917 days	3.56%	3.27%
**House 4**							
17	Refrigerator	Liebherr KTS14*	2017-04-17 19:33:30	2020-11-16 09:32:07	1308 days	1.02%	0.78%
18	Freezer	AEG arctis	2017-04-17 19:15:04	2020-12-09 09:59:13	1331 days	0.68%	0.44%
19	Dish Washer	Bosch	2017-04-17 19:51:27	2020-12-09 09:00:00	1331 days	0.46%	0.22%
20	Washing Machine	AEG Lavamat Exclusive 54569	2017-04-17 19:17:14	2020-12-09 09:00:00	1331 days	0.78%	0.31%
**House 5**							
5	Dish Washer	Bosch SMS69N48EU	2016-08-10 23:26:28	2019-01-18 08:00:00	890 days	32.59%	12.78%
6	Washing Machine	Miele SOFTTRONIC W2241	2016-08-16 10:32:18	2019-01-17 17:08:06	884 days	21.39%	10.25%
8	Office Desk		2016-12-06 12:58:02	2019-01-18 08:00:08	772 days	4.81%	4.44%
9	Refrigerator	Bauknecht	2016-08-10 14:10:32	2019-01-18 08:00:00	890 days	10.40%	8.23%
30	Coffee Machine	Bezzera BZ09	2017-06-28 17:26:00	2019-01-18 08:00:00	568 days	6.19%	1.06%
**House 6**							
31	Dish Washer		2017-07-22 09:00:03	2018-02-01 23:00:00	194 days	5.27%	5.24%
32	Refrigerator		2017-07-22 08:54:03	2018-02-01 23:00:00	194 days	5.24%	5.21%
33	Washing Machine	Miele Novotronic W1514	2017-07-22 09:16:03	2017-08-12 17:00:00	21 days	0.00%	0.00%
34	Dryer	Miele Novotronic T7644C	2017-07-22 09:13:02	2018-02-01 16:00:00	194 days	5.34%	5.27%
36	Dish Washer	Siemens Extrakl. Festival Spuler	2017-07-26 21:20:02	2018-02-01 23:00:00	190 days	3.65%	3.62%
37	Refrigerator	Haier HEC MCS662FIX	2017-07-26 21:11:03	2018-03-05 10:00:00	221 days	4.63%	4.60%
**House 7**							
68	Washing Machine	Miele Hydromatic W701	2017-10-08 15:01:04	2020-12-09 09:40:09	1157 days	29.35%	29.12%
69	Other	Whirlpool	2017-10-08 15:01:04	2018-07-20 08:05:50	284 days	35.06%	34.14%
70	TV	Sony KDL-48W605B	2017-10-08 15:01:04	2020-05-04 11:00:07	938 days	14.13%	13.88%
71	Coffee Machine	Saeco Magic Comfort +	2017-10-08 16:00:00	2020-12-09 10:00:00	1157 days	29.38%	29.12%
**House 8**							
24	Washing Machine	Miele W 5873 WPS Edition 111	2017-06-06 15:29:23	2018-07-28 08:00:12	416 days	1.13%	1.01%
26	Dish Washer		2017-06-18 13:47:12	2018-07-28 08:00:00	404 days	1.13%	1.04%
27	Coffee Machine	Bezzera Mitica Top MN	2017-06-18 13:37:34	2018-07-28 08:00:12	404 days	1.04%	1.03%
28	Office Desk		2017-06-18 14:13:03	2018-07-28 08:00:13	404 days	1.09%	1.03%
35	Refrigerator		2017-07-23 20:26:03	2018-07-28 08:00:13	369 days	0.01%	0.00%
51	Smart Meter Phase	Modbus Smart Meter Phase 1	2017-09-05 15:45:29	2018-07-28 08:00:00	325 days	0.28%	0.00%
52	Smart Meter Phase	Modbus Smart Meter Phase 2	2017-09-05 17:53:03	2018-07-28 08:00:00	325 days	1.83%	1.38%
53	Smart Meter Phase	Modbus Smart Meter Phase 3	2017-09-05 17:55:03	2018-07-28 08:00:00	325 days	9.71%	9.38%
59	Smart Meter Total	Modbus Smart Meter Total	2017-09-12 14:10:03	2018-07-28 08:00:00	318 days	13.97%	13.58%
**House 9**							
44	Dish Washer		2017-08-05 18:13:18	2019-02-03 10:09:47	546 days	5.86%	5.52%
45	Refrigerator		2017-08-05 18:07:03	2020-03-17 08:00:07	954 days	2.98%	2.92%
46	Washing Machine		2017-08-05 18:13:30	2020-03-17 08:00:00	954 days	2.99%	2.93%
**House 10**							
65	Refrigerator		2017-09-20 16:04:04	2019-11-01 13:00:00	771 days	8.13%	7.62%
66	Dish Washer		2017-09-20 17:00:00	2019-11-01 13:00:00	771 days	8.18%	7.63%
67	Washing Machine		2017-09-20 16:07:55	2019-11-01 13:00:00	771 days	8.14%	7.62%
**House 11**							
38	Dryer		2017-07-27 20:37:35	2017-12-15 09:00:00	140 days	0.28%	0.00%
**House 12**							
61	Washing Machine		2017-09-13 16:55:35	2018-07-31 11:00:00	320 days	3.29%	3.29%
62	Dish Washer		2017-09-13 16:50:13	2018-07-31 11:00:00	320 days	3.27%	3.27%
63	Dryer		2017-09-13 17:00:00	2018-07-31 11:00:00	320 days	3.30%	3.30%
64	Refrigerator		2017-09-13 16:50:04	2018-07-31 11:00:10	320 days	3.28%	3.28%
**House 13**							
39	Washing Machine		2017-07-30 10:56:08	2018-10-06 11:00:05	433 days	7.59%	7.56%
40	Dish Washer		2017-07-30 10:30:38	2018-10-06 11:00:09	433 days	3.00%	3.00%
41	Refrigerator		2017-07-30 10:58:59	2018-10-06 11:00:10	433 days	4.73%	4.73%
**House 14**							
81	Other	Heat Pump	2017-10-24 11:44:22	2019-10-14 16:06:02	720 days	19.08%	19.05%
82	Refrigerator		2017-11-12 14:58:03	2020-12-09 09:59:59	1122 days	10.87%	10.51%
83	Washing Machine		2017-11-12 15:49:05	2020-12-09 09:40:16	1122 days	10.54%	10.52%

The columns “Missing >1 h 5 sec” and “Missing >1day” provide an indicator for missing data, defined as the sum of gaps in relation to the duration.

**Fig. 4:**
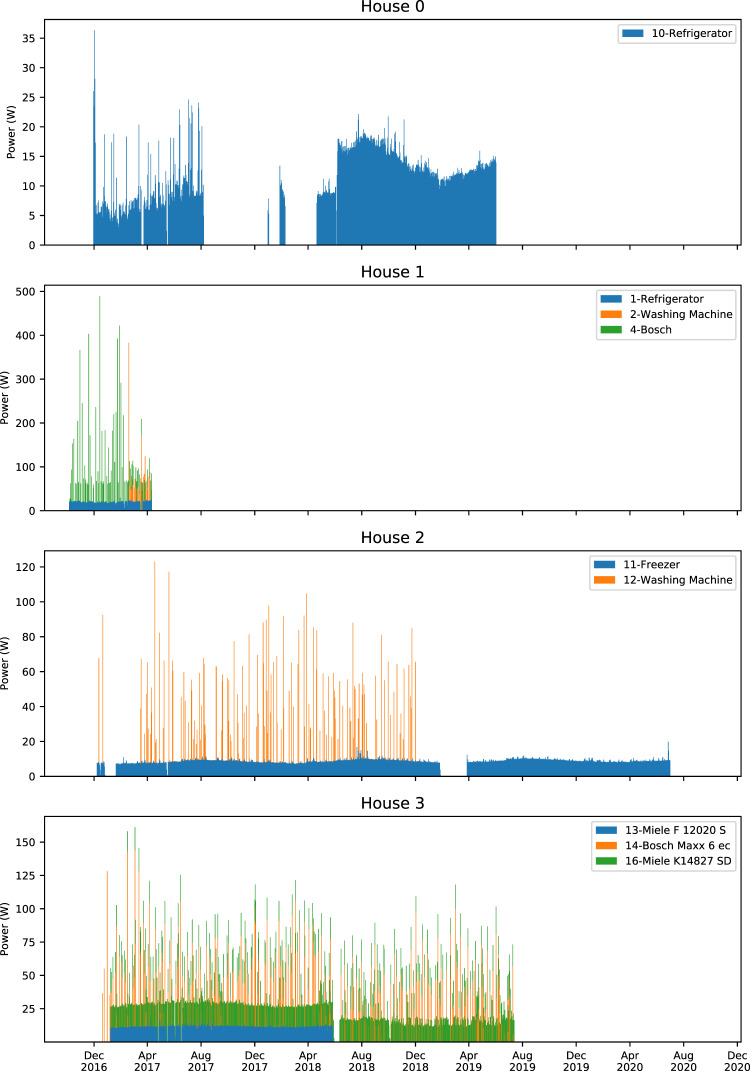
Stacked average daily power demand of monitored appliances in Houses 0–3. X-axes ranges are aligned, Y-axes are not aligned to increase per house readability. Each color block is stacked and shows daily average power demand of an appliance. Periods where there is no power demand of a certain device may be caused by monitoring interruptions as well as not using the appliance.

**Fig. 5:**
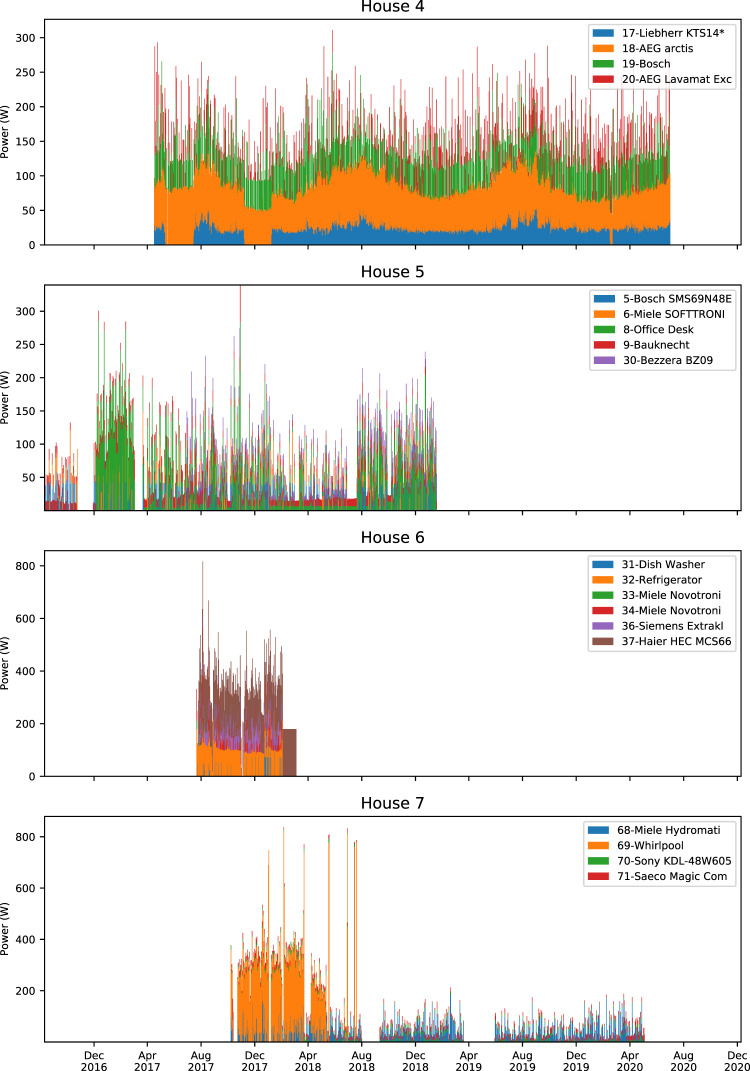
Stacked average daily power demand of monitored appliances in Houses 4–7. X-axes ranges are aligned, Y-axes are not aligned to increase per house readability. Each color block is stacked and shows daily average power demand of an appliance. Periods where there is no power demand of a certain device may be caused by monitoring interruptions as well as not using the appliance.

**Fig. 6:**
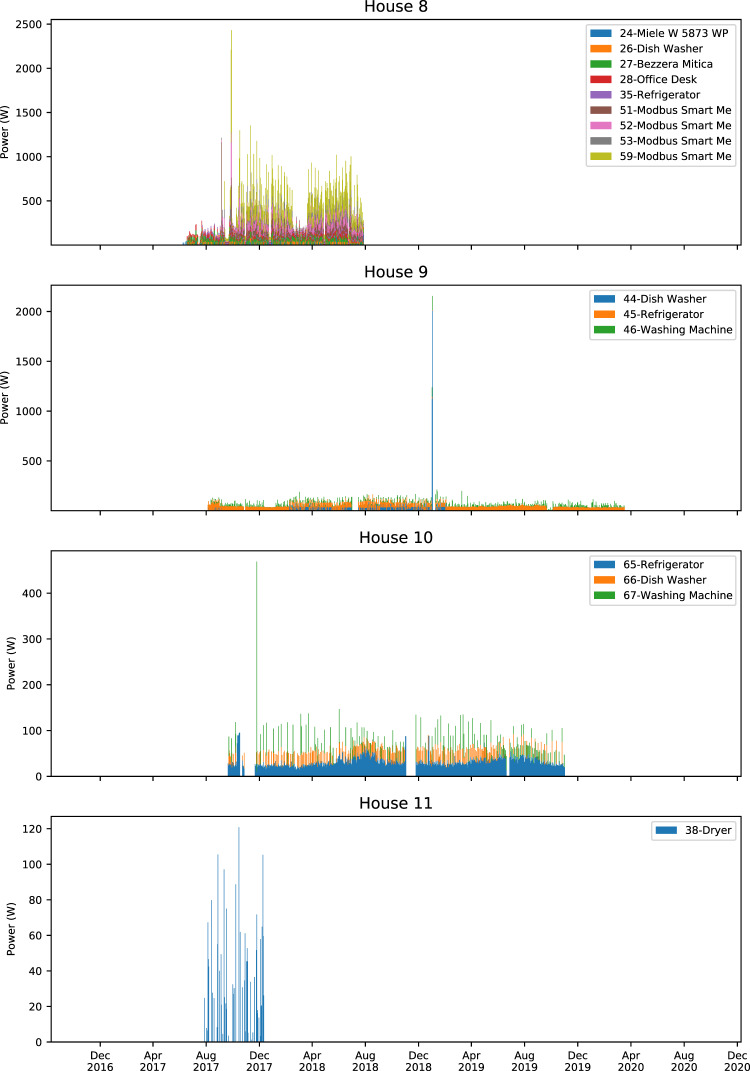
Stacked average daily power demand of monitored appliances in Houses 8–11. X-axes ranges are aligned, Y-axes are not aligned to increase per house readability. Each color block is stacked and shows daily average power demand of an appliance. Periods where there is no power demand of a certain device may be caused by monitoring interruptions as well as not using the appliance.

**Fig. 7:**
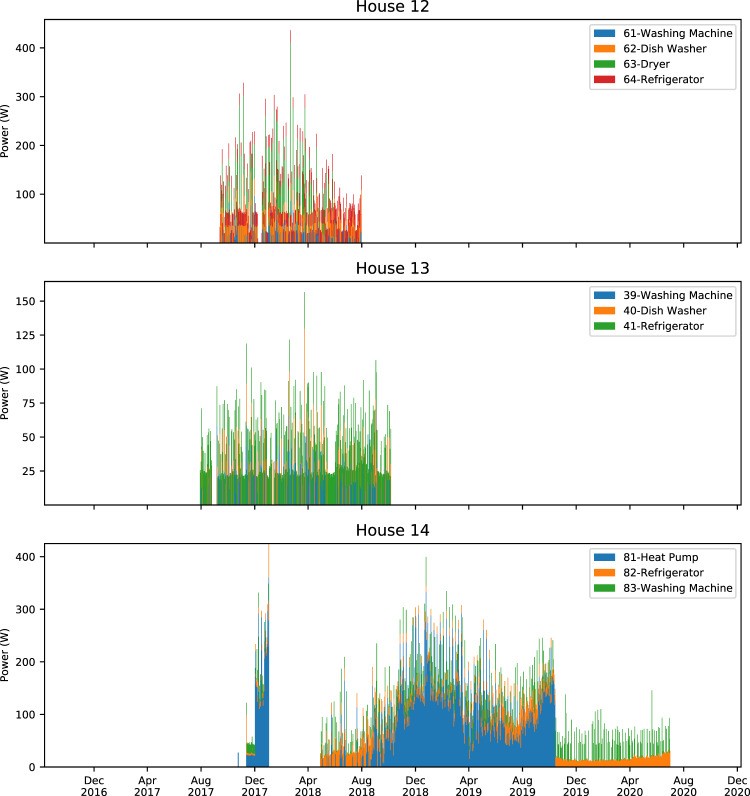
Stacked average daily power demand of monitored appliances in Houses 12–14. X-axes ranges are aligned, Y-axes are not aligned to increase per house readability. Each color block is stacked and shows daily average power demand of an appliance. Periods where there is no power demand of a certain device may be caused by monitoring interruptions as well as not using the appliance.

Figures 4–7 show stacked daily average consumption for all houses. The aligned charts show the periods the data was recorded and can be used for visual examination of missing data or analyzing seasonal changes wherein, for example, refrigerators require more electricity during summer periods.

For 14 cycle based appliances we provide manual annotations. These annotations provide start and stop timestamps of a cycle; there are multiple labels per appliance where possible in order to annotate different modes. For example, for the refrigerator with item ID 10 in house 0, we provide separate annotations for light and compressors. Thus, the annotations can be used as ground-truth for appliance usage predictions which, to our best knowledge, does not exist for any long-term electricity dataset available. This will allow training and evaluation of classifiers that can detect “usage” of the refrigerator based on the light being switched on when the door is opened. Additionally, we provide demographic data for each household’s residents such as age, absence duration and regularity of absence.

The DEDDIAG dataset is open-access and is hosted on Figshare^28^.

### Structure

All data are provided as plain text, tab-separated value files (TSV). There is one directory per house (house_00/, house_01/,) containing a house.tsv file with the house description. The description provides demographic data such as number of residents, their age, regularity of absence and normal absence duration. Appliances meta data is provided in items.tsv, containing a unique ID, name, device category and house ID. Appliance categories provide a grouping label. Available categories are: Refrigerator, Freezer, Washing Machine, Dryer, Dish Washer, Coffee Machine, TV, Office Desk, Smart Meter Phase, Smart Meter Total and Other.

The measurements, annotation and annotation labels of each appliance are provided in separate files:

- item_XXXX_data.tsv.gz,
- item_XXXX_annotations.tsv,
- item_XXXX_annotation_labels.tsv.

The measurement files are each compressed using gzip to reduce size. The data is split per house and appliance in order to be able to work on a single home or appliance. While the tsv files can easily be used directly, an import bash script called import.sh is provided to automatically import the data into a docker-based PostgreSQL database. Detailed instructions are provided as part of the dataset archive in README.md. The compressed dataset has a size of 14 GB, the uncompressed, imported data requires about 140 GB. All measurements are stored in a sparse manner where only value changes are recorded. In order to be able to detect connectivity problems of the metering device, there is at least one reading per hour. Therefore, if the time span between two measurements is greater than 1 h 5 sec, it should be assumed that there are missing values. A measurement record is stored as <item_id> <time> <value> where <item_id> corresponds to an ID in items.tsv. <time> is a UTC datetime string using the YYYY-MM-DD HH:MI:SS.US format, measurements are stored as float values.

Annotations are provided as <id> <item_id> <label_id> <start_date> <stop_date> where start, stop, are UTC datetime strings using the YYYY-MM-DD HH:MI:SS format. Annotations are only provided to full seconds, thus the corresponding measurement can be found using the provided custom SQL function round_timestamp().

## Technical Validation

All parts of the data collection system were tested in a lab environment. The collected measurements were captured as provided by the metering device. The mains meter was certified to be MID (Measuring Instruments Directive) calibrated, the appliance-level meters were not. A lab test on the wireless appliance meter devices was performed to assure constant readings over different metering devices. Another lab test was performed to compare readings of the appliance and mains meters, showing that the appliance meters systematically provided 2% to 4% lower readings compared to the mains meters.

### Sample rate precision

Although there is one reading per second, the exact rate varies due to the technical limitations of the metering device. For single appliance measurements the rate will usually fluctuate between 0.9 Hz and 1.1 Hz. The timestamp precision is sub-second, rounding to full seconds may result in having two measurements for the same second. The recommended handling for such cases is to only keep the later value to avoid changing time-order of the two values, since this may introduce a significant error considering only value changes are recorded.

### Missing values

There are missing values within the dataset. In order to be able to detect failures, the system recorded at least one value per hour per appliance and therefore time gaps that are greater than 1 h 5 sec must be treated as missing values. The additional 5 seconds are added as a fair dealing gap because small deviations from the 1 h are of no significance as these gaps only represent periods where the electricity demand has not changed, which in reality only applies to 0 Watt cases. An overview of monitoring gaps is given in Table 1 as sum of missing periods relative to the total duration. There are two columns, one for a gap size of more than 1 h 5 sec, and another for a gap size of more than one day, where the latter must always be smaller. The >1 h 5 sec missing data ranges from 0.28% on item 38 to 59.40% on item 4, and the >1 day missing data ranges from 0.00% to 49.26% for the same appliances.

As an example Table 2 provides a more detailed insight into monitoring gaps of item 4 and item 69 that have an overall >1 h 5 sec missing data of 59.40% and 35.06%. The items were chosen as an example since they have very different missing data patterns. The table shows how much of the missing data falls within a certain gap length, clearly indicating that item 69 suffers from long term interruptions where 89% of the missing data are from gaps >5 days where there is no gap of that length for item 4. Item 69 has a single monitoring gap of about 33 days, accounting for 34% of its missing data, while the largest gap for item 4 is 4 days.

**Table 2:** Comparison of available data of item 69 and 4 as an example for missing data caused by interruption of the monitoring.

Gap Length	Item 69	Item 4
>1 hours	100.0%	100.0%
>2 hours	100.0%	97.5%
>3 hours	100.0%	94.5%
>4 hours	100.0%	92.7%
>5 hours	100.0%	92.4%
>6 hours	100.0%	92.2%
>1 days	97.4%	82.9%
>1 days 12 hours	97.4%	64.4%
>2 days	97.4%	50.0%
>3 days	97.4%	12.6%
>4 days	94.0%	3.9%
>5 days	89.3%	0.0%
>33 days	34.0%	0.0%

The table shows how much of the missing data is of certain length, illustrating the differences of missing data. 89.3% of the missing data of item 69 come from interruptions >5 days, meaning there are mostly long term interruptions. 50% of missing data on item 4 come from interruptions >2 days, meaning there are mostly short term interruptions.

As the analysis as well as impacts of the missing data depend on the application of the dataset, detailed inspections of missing data must be performed when using the dataset. The DEDDIAG -loader software library described in section Code Availability provides a method to find missing periods to assist analysis. Missing data can also be seen visually in the daily average power demand, Figs. 4–7.

## Usage Notes

The dataset release is accompanied by a full usage description. The dataset has not been preprocessed and is provided as recorded. It is advised to read the README.md for detailed usage instructions. All data is provided as plain-text TSV files. Although we recommend and provide information to import all data into a PostgreSQL database, the plain-text data can be parsed directly with standard tooling.

## Baseline Results

In addition to the dataset we provide baseline results for appliance category identification and automatic event annotations.

### Appliance category identification

The dataset is used to train and evaluate an appliance category classifier. Such a classifier can be used to identify single appliance recordings of unknown appliances and therefore act as the first classifier for further tasks such as event annotation or usage prediction. The task is performed on all available appliances using the unaligned recordings, meaning that the classifier has to be able to perform on a randomly chosen segment. The sparse recorded data is taken as is, which prevents trying to classify long switched off states where the power demand is 0 Watt.

As a baseline we train a k-nearest neighbors classifier for all available categories: Coffee Machine, Dish Washer, Dryer, Freezer, Office Desk, Other, Refrigerator, TV, Washing Machine.

The classifier is trained using a sliding window where the window size will also define the recording length required to identify an appliance. With a sample rate of 1 Hz, the window size represents the number of seconds the appliance needs to run in order to identify it. In order to reduce the feature space dimensionality, we extract window-based features using the window’s mean as well as coefficients of a discrete wavelet transform. The mean value provides a good indicator as appliances are composed of very distinct power demand groups. The wavelet transform is very common in time series classification and provides a low dimensional representation of the signal. It is performed as a multi-level decomposition using the Daubechies kernel db1. The decomposition level is set such that we get a total of four components, two detail and two approximation coefficients, making the feature space independent of the window size and therefore removing the problem of exploding dimensionality for large window sizes. Combined with the window’s mean, this results in a five-dimensional feature vector, which is normalized and standardized independently based on the training data.

The classification is performed using the k-nearest neighbor algorithm, a very robust and commonly used classifier with the capability of separating non-linear class boundaries based on a distance metric. We separate based on 5 neighbors using the Minkowski distance. 5 million values per category are used in order to reduce the total data size while still having a significant number of samples, taking equal numbers from each appliance in case multiple appliances are present for a category. The evaluation is performed using a 5-fold cross-validation for window sizes 2^*n*^ with $n\in [2,11]$ and step size 3.

Table 3 shows the *F*_1_-score per class and the weighted average over all classes for tested window sizes. The *F*_1_-score is calculated as:

1 $${F}_{1}=2\cdot \frac{{\rm{precision}}\cdot {\rm{recall}}}{{\rm{precision}}+{\rm{recall}}}=\frac{{\rm{TP}}}{{\rm{TP}}+0.5({\rm{FP}}-{\rm{FN}})},\quad {\rm{precision}}=\frac{{\rm{TP}}}{{\rm{TP}}+{\rm{FP}}},\quad {\rm{recall}}=\frac{{\rm{TP}}}{{\rm{TP}}+{\rm{FN}}},$$

where TP, FP and FN are the number of true positives, false positives, and false negatives.

**Table 3:** Baseline result of appliance category identification using k-nearest neighbors classification.

Window Size	4	8	16	32	64	128	256	512	1024	2048
Coffee Machine	0.9137	0.9193	0.9160	0.9214	0.9404	0.9412	0.9304	0.9348	0.9434	0.9552
Dish Washer	0.5163	0.5735	0.6452	0.7193	0.7878	0.8391	0.8634	0.8782	0.8930	0.9113
Dryer	0.8457	0.8542	0.8571	0.8714	0.8901	0.9083	0.9129	0.9293	0.9413	0.9433
Freezer	0.8002	0.8198	0.8391	0.8576	0.8803	0.9042	0.9052	0.9194	0.9468	0.9767
Office Desk	0.7295	0.7567	0.7824	0.8024	0.8219	0.8473	0.8549	0.8844	0.9274	0.9500
Other	0.8517	0.8575	0.8639	0.8685	0.8776	0.8893	0.9041	0.9198	0.9358	0.9556
Refrigerator	0.7010	0.7239	0.7534	0.7857	0.8178	0.8466	0.8675	0.8987	0.9293	0.9504
TV	0.7581	0.8222	0.8725	0.9020	0.9189	0.9335	0.9415	0.9465	0.9556	0.9663
Washing Machine	0.6232	0.6736	0.7295	0.7801	0.7876	0.7904	0.8003	0.8194	0.8420	0.8702
Weighted Average	0.7443	0.7743	0.8043	0.8326	0.8566	0.8764	0.8855	0.9023	0.9231	0.9421

Results show *F*_1_-score for each category at tested window sizes as well as the weighted average over all classes based on a 5-fold cross-validation. Results strongly indicate that *F*_1_-score increases with increasing window size.

The results indicate that the *F*_1_-score increases with increasing window size, which means that although the number of features presented to the k-nearest neighbor algorithm are independent from the window size, the extracted window features contain more distinct class boundaries when using a larger window size. For very small window sizes, the Coffee Machine category already performs very well with a score >0.91 even for window size 4. The best performing category at large window sizes is Freezer, with a maximum score of 0.9767. While at window size 2048 nearly all categories perform well with scores >0.90, the Washing Machine performs considerably worse with a score of 0.87. Washing Machines and Dish Washers are the most difficult appliances to distinguish, as they both present the highest miss-classification class for each other.

### Event annotation

Event annotation, also known as event detection, is the task of finding events in electricity data. There have been numerous publications on event detection with a NILM background. These event detection tasks are usually performed using thresholding methods^29^. Since there are no public datasets that provide ground truth, the evaluation of most publications in the essence do not predict events, but rather the presence of electricity consumption. DEDDIAG provides manual event annotations that can act as ground truth to train and evaluate event annotation algorithms. Appliance usage prediction and user behavior algorithms, such as^25,30^, will benefit from more advanced event annotations that are evaluated against manual annotated ground truth, as theses algorithms require event annotations as their ground truth.

We denote event annotation as a segmentation task where an event segment $e=[{t}_{0},{t}_{1}]$ is defined by the interval between two timestamps ${t}_{0}$, ${t}_{1}$, ${t}_{0} < {t}_{1}$ during which an appliance is running, e. g. a washing machine being started at ${t}_{0}$, finishing a full washing cycle at ${t}_{1}$ (note that the appliance might consume only a very small amount of power at certain times while running, which may make it very hard to distinguish this case from the actual end of the cycle). For most appliances, such as washing machine and dishwasher, there are no overlapping segments. This is also the case if there are different labels defined for one appliance, e. g., PreWash and Normal. For refrigerators and freezers, overlap between the light and compressor labels are possible, but overlap within one label cannot occur.

**Fig. 8:**
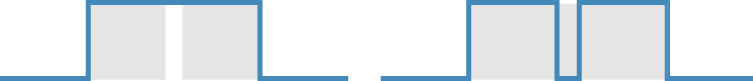
False annotations that are very mildly punished when evaluating per timestamp using *F*_1_-score or similar. The blue line represents the real event segment, the gray area shows the predicted event segment. On the left the real event segment is predicted as two segments, and on the right two real event segments are predicted as a single one. In both cases the Jaccard-Time-Span-Event-Score (JTES) will result in a score ≤0.5.

Since in a NILM context events are defined by a single timestamp, the evaluation of such events is usually done based on correctness of the label for each time-step using standard scores such as true positive rate, false positive rate, accuracy or *F*_1_. For usage analysis or event attribution these scores are problematic because all such events are extremely rare, especially when only annotating the start or end. To put it in perspective: an appliance that only is switched on once per day will, in a dataset recorded with 1 Hz, result in having to find one value within 86400 data points. Evaluating an appliance based on ON/OFF status for each available time-step will for long-running appliances result in a much more balanced task. While for electricity attribution tasks such as disaggregation this evaluation can be seen as fair, for appliance usage analysis it is still not strict enough. A per timestamp evaluation will not provide strong enough punishment for many unwanted annotations such as splitting or combining of event segments as shown in Fig. 8.

For usage analysis a split or combined event segment will result on wrong overall event counts, wrong usage lengths and thus a false analysis of usage behavior. We therefore introduce a new score called Jaccard-Time-Span-Event-Score (JTES) that is based on the Intersection-over-Union (IoU) metric, also known as Jaccard index. The Jaccard index computed on a single segment is:

2 $${\rm{iou}}({e}_{{\rm{T}}},{e}_{{\rm{P}}})=\frac{Intersection}{Union}=\frac{\min ({t}_{{\rm{T1}}},{t}_{{\rm{P1}}})-\max ({t}_{{\rm{T0}}},{t}_{{\rm{P0}}})}{\max ({t}_{{\rm{T0}}},{t}_{{\rm{P0}}},{t}_{{\rm{T1}}},{t}_{{\rm{P1}}})-\min ({t}_{{\rm{T0}}},{t}_{{\rm{P0}}},{t}_{{\rm{T1}}},{t}_{{\rm{P1}}})},$$

where ${e}_{{\rm{T}}}$ is the true event segment and ${e}_{{\rm{P}}}$ the predicted one; this basically assumes that a box of height one is used for comparing regions. The Jaccard index is widely used to evaluate image segmentation tasks such as object detection, where commonly true positives are defined as having an overlap of more than 50%^6^, which are then used to calculate an overall accuracy score. The JTES score avoids setting an arbitrary threshold and therefore eliminates the drawbacks of using pure IoU. In a first step we compute an average IoU for each true event segment, only taking into account predictions that actually have at least a partial overlap with the true event segment. Let ${e}_{{\rm{T}}i},i=0,\ldots ,{N}_{{\rm{T}}}-1$ be a true event segment and ${e}_{{\rm{P}}j}$, $j=0,\ldots ,{N}_{{\rm{P}}}-1$ a predicted segment, where ${N}_{{\rm{T}}},{N}_{{\rm{P}}}$ is the number of true and predicted segments, respectively. The average IoU ${A}_{i}$ for true event *i* is then given by

3 $${A}_{i}=\frac{1}{{N}_{{\rm{NZ}}i}}\mathop{\sum }\limits_{j=0}^{{N}_{{\rm{P}}}-1}{\rm{iou}}({e}_{{\rm{T}}i},{e}_{{\rm{P}}j}),$$

where ${\rm{iou}}({e}_{{\rm{T}}i},{e}_{{\rm{P}}j})$ computes the IoU for the segments ${e}_{{\rm{T}}i}$ and ${e}_{{\rm{P}}j}$ as defined in (2), and ${N}_{{\rm{NZ}}i}$ is the number of non-zero IoU-values in the sum (i. e. the predictions with an overlap). The final score is then calculated by summing all *A*_*i*_ and normalization to the number of true event segments *N*_T_ corrected by the number of false positive predictions *N*_FP_:

$${\rm{JTES}}({{\boldsymbol{e}}}_{{\rm{T}}},{{\boldsymbol{e}}}_{{\rm{P}}})=\frac{\mathop{\sum }\limits_{i=0}^{{N}_{{\rm{T}}}}{A}_{i}}{{N}_{{\rm{T}}}+{N}_{{\rm{FP}}}},\hspace{7.5pt}{{\boldsymbol{e}}}_{{\rm{T}}}=\left({e}_{{\rm{T}}0},{e}_{{\rm{T}}1},\ldots ,{e}_{{\rm{T}}{N}_{{\rm{T}}}-1}\right),\hspace{7.5pt}{{\boldsymbol{e}}}_{{\rm{P}}}=\left({e}_{{\rm{P}}0},{e}_{{\rm{P}}1},\ldots ,{e}_{{\rm{P}}{N}_{{\rm{P}}}-1}\right).$$

The JTES score requires non-overlapping true event segments, i. e. ${e}_{{\rm{T}}i}\cap {e}_{{\rm{T}}j}={\rm{0}}\,/\;\forall i,j,i\ne j$. It is designed to reflect real-world expectations on event annotation algorithms, where an event that is split in half will at best only be evaluated as half correct, the same applies to scenarios where two true events have been merged into one event as shown in Fig. 8. Splitting a real event in two successive events will result in a JTES score of at most 0.5, while common scores such as accuracy or *F*_1_ will evaluate each time step independently and still result in perfect scores.

The principles of JTES are:

- Score is in range [0, 1], where 0 is lowest and 1 best,
- false positives and false negatives are equally bad,
- if a true event spans over multiple predicted events, the result is averaged,
- if the predicted event spans over multiple true events, the result is accounted for accordingly,
- if ${N}_{{\rm{T}}}=0$ and ${N}_{{\rm{P}}} > 0$, the score is 0,
- if ${N}_{{\rm{T}}} > 0$ and ${N}_{{\rm{P}}}=0$, the score is 0,
- if ${{\boldsymbol{e}}}_{{\rm{T}}}={{\boldsymbol{e}}}_{{\rm{P}}}$ (assuming the segments are ordered by start time), the score is 1.

As a baseline algorithm we present results for a simple lower bound thresholding algorithm. The appliance is seen as switched-on when the average electricity demand *E* in a sliding window *w* reaches a threshold *t*:

4 $$E(w)=\left\{\begin{array}{cc}0 & {\rm{avg}}(w) < t\\ 1 & {\rm{avg}}(w)\ge t\end{array}\right..$$

The threshold is defined as the non-zero minimum average window calculated over each event in the train split.

We use a 5-fold cross-validation and test window sizes in range 1–24. Table 4 lists results for 15 segmentation tasks, where the best window was chosen based on the highest JTES score. The results clearly show that the fridge compressor cycles can reliably be annotated having a JTES of 0.8817, 0.8855, and 0.9348. The fridge light however, having very low JTES scores of 0.1589, 0.2713, and 0.0403, cannot be annotated correctly, which can be explained by the lack of an upper bound that would allow distinguishing compressor and light power. It also shows the problem when evaluating rare events using an *F*_1_-score, as while the JTES on the light of item 10 is only 0.0403, the *F*_1_-score is at 0.95, which would indicate that the algorithm performs well. The light and compressor can be present at the same time, which would require a second lower bound threshold, if even possible using a simple threshold approach. Washing machines show very different JTES scores, ranging from 0.0385 to 0.6055, where especially item 6 with the lowest JTES score still has a *F*_1_-score of 0.9196. The algorithm had to choose a very low threshold as there are many periods where the washing machine uses very little electricity which then splits the event into multiple parts, which is heavily punished by the JTES, but not by the *F*_1_-score. Dish washers show similar results, having JTES scores ranging from 0.0817 to 0.7167 while having *F*_1_-scores ranging from 0.8722 to 0.9959. The very bad JTES score on the washing machine with item 6 and dish washer with item 26 can be explained by a very high number of false positives of short length, that are very mildly punished by the *F*_1_-score. This in particular demonstrates the weaknesses of evaluating based on single timestamps instead of a time span and highlights the importance of the novel JTES score.

**Table 4:** Baseline result of event annotation task using a thresholding method.

Item	Category	Labels	Window Size	JTES	*F*_1_
1	Refrigerator	33 (C)	13	0.8817	0.9967
1	Refrigerator	34 (L)	1	0.1589	0.7960
2	Washing Machine	35, 36	24	0.6055	0.9994
4	Dish Washer	17, 18	22	0.7167	0.9959
5	Dish Washer	9, 10, 15	1	0.2370	0.9958
6	Washing Machine	5, 21, 26, 25	24	0.0385	0.9196
9	Refrigerator	31 (C)	7	0.8855	0.9967
9	Refrigerator	32 (L)	1	0.2713	0.8089
10	Refrigerator	29 (L)	1	0.0403	0.9530
10	Refrigerator	30 (C)	5	0.9348	0.9989
12	Washing Machine	37	24	0.4587	0.9768
19	Dish Washer	2, 4, 19, 24	1	0.4607	0.9370
20	Washing Machine	6, 7, 8, 11, 40, 41	1	0.4974	0.9737
24	Washing Machine	14	1	0.3796	0.9818
26	Dish Washer	12, 22, 23	24	0.0817	0.8722

While the task for dish washers and washing machines does not distinguish between different labels per item, for refrigerators the two labels compressor (C) and light (L) are evaluated as two separate tasks. Results are evaluated using Jaccard-Timespan-Event-Score (JTES) and *F*_1_-score. Result is shown for window sizes where JTES was highest in a trial with sizes ranging from 1 to 24.

Overall, using a simple thresholding algorithm will not provide a good and reliable event detection algorithm and the simple approach would benefit from merging small successive predictions as well as filtering based on event length.

## Data Availability

The full data collection system is published under MIT license and is available under https://DEDDIAG.github.io. The dataset itself is published as tab-separated text files together with code to import all data into a PostgreSQL instance. An SQL function called get_measurements() is provided to get seconds-based measurements, where readings are converted from value-changes to seconds-based readings using interpolation; timestamps are rounded to nearest seconds. There is also a python package available https://github.com/DEDDIAG/DEDDIAG-loader.git that assists in retrieving data into a pandas-DataFrame/numpy-array.
